# Transcriptome analysis provides insights into xylogenesis formation in Moso bamboo (*Phyllostachys edulis*) shoot

**DOI:** 10.1038/s41598-018-21766-3

**Published:** 2018-03-02

**Authors:** Hui Zhang, Ye-qing Ying, Jie Wang, Xian-hai Zhao, Wei Zeng, Cherie Beahan, Jun-bo He, Xiao-yang Chen, Antony Bacic, Li-li Song, Ai-min Wu

**Affiliations:** 1 State Key Laboratory of Subtropical Silviculture, School of Forestry and Biotechnology, Zhejiang A&F University, Lin’an, 311300 Zhejiang Province, People’s Republic of China; 2 Guangdong Key Laboratory for Innovative Development and Utilization of Forest Plant Germplasm, Guangzhou, 510642 China; 3 College of Forest, South China Agricultural University, Guangzhou, 510642 China; 4 ARC Center of Excellence in Plant Cell Walls, School of BioSciences, the University of Melbourne, Parkville, VIC 3010 Australia

## Abstract

Maturation-related changes in cell wall composition and the molecular mechanisms underlying cell wall changes were investigated from the apical, middle and basal segments in moso bamboo shoot (MBS). With maturation extent from apical to basal regions in MBS, lignin and cellulose content increased, whereas heteroxylan exhibited a decreasing trend. Activities of phenylalanine amonnialyase (PAL), cinnamyl alcohol dehydrogenase (CAD) and cinnamate-4-hydroxylase (C4H), which are involved in lignin biosynthesis, increased rapidly from the apex to the base sections. The comparative transcriptomic analysis was carried out to identify some key genes involved in secondary cell walls (SCW) formation underlying the cell wall compositions changes including 63, 8, 18, and 31 functional unigenes encoding biosynthesis of lignin, cellulose, xylan and NAC-MYB-based transcription factors, respectively. Genes related to secondary cell wall formation and lignin biosynthesis had higher expression levels in the middle and basal segments compared to those in the apical segments. Furthermore, the expression profile of *PePAL* gene showed positive relationships with cellulose-related gene *PeCESA4*, xylan-related genes *PeIRX9* and *PeIRX10*. Our results indicated that lignification occurred in the more mature middle and basal segments in MBS at harvest while lignification of MBS were correlated with higher expression levels of *PeCESA4*, *PeIRX9* and *PeIRX10* genes.

## Introduction

Moso bamboo (*Phyllostachys edulis*), belonging to the subfamily *Bambusoideae* of the *Poaceae* family, is the most ecologically and economically important and culturally popular bamboo species in the world^1^. Moso bamboo shoot (MBS), widely grown in Southern and Eastern China, is a young, tender stalk emerging from nodes of the (pseudo-)rhizome of bamboo plants. The edible part of MBS consists of meristematic cell tissue with regions of rapid cell division and differentiation, which is enveloped in protective, non-edible leaf sheaths. As a commercially important shoot producing species, MBS has increasingly become popular for its rich dietary fibre and distinctive flavor^2^. However, due to its rapid flavor loss and texture deterioration after harvest, fresh MBS is mostly been distributed locally as the food quality decreases dramatically during long distance transportation. One of the most important contributing factors to texture is the cell wall, which consists of cellulose microfibrils embedded in a matrix of pectins, hemicelluloses, proteins and lignin. MBS undergoes a process of lignifying, which proceeded progressively from the tender immature apical to rigid base part both at harvest and during the maturation and development process, making them rigid and woody and resulting in a loss of gustative quality^3,4^. Thus, analyzing increased lignin depositions from the top to bottom may reflect the lignification of harvested bamboo shoot, therefore being helpful to understand molecular mechanisms of post-harvest physiology.

Plant cell wall can be divided into primary (PCW) and secondary cell walls (SCW), which PCW is the wall of growing cells and SCW is formed after growth has stopped and differentiation has started. Unlike hardwood and softwood trees, such as *populus*, the development of which include primary and secondary thickening growth^5^, bamboo developed only primary shoot, which occurred in vascular bundles embedded in parenchymatous ground tissue^6^. As ‘developmentally immature, rapidly growing’ subterraneous tissues, bamboo shoot underwent the lignification of protoxylem vessels in the early stage of vascular bundle differentiation, and subsequently metaxylem vessel and fibre walls from the middle lamella and cell corners, finally SCW deposition formed^7^. It was shown that when SCW is deposited, all the biosynthesis genes for cellulose, hemicellulose and lignin need to be up-regulated. In addition, the expression level of genes responsible for the supply of associated nucleotide sugars, phenylpropanoid pathway precursors, methyl and acetyl donors and many other secondary wall biosynthetic pathway precursors need increase as well^8^. Therefore, SCW formation is related with up-regulation of signal pathway genes. For example, functional molecular and co-expression analysis of *IRX* genes led to the identification of SCW-specific cellulose synthase subunit A (*CesA*) genes (*IRX1*/*CesA8*, *IRX3*/*CesA7*, and *IRX5*/*CesA4*), SCW-specific hemicellulose biosynthetic genes (*IRX7*, *IRX8*, *IRX9*, *IRX10*, *IRX14*, and *IRX15*), and lignin biosynthetic genes (*IRX4* and *IRX12*)^9–15^. Additionally, SCW deposition is regulated by a complex network of transcription factors^16^, including a group of NAC domain-containing transcription factors. This group includes members like SCW-associated NAC domain protein 1 (SND1)^17^, NAC secondary wall thickening-promoting factors (NST1 and NST2)^18^, and vascular-related NAC domain factors (VND6 and VND7)^19^, which are master regulators of SCW deposition. Currently, candidate genes of model and non-model species involved in the biosynthesis of SCWs have mostly been investigated in some woody and herbaceous model species^20,21^. However, in some harvested fruits and vegetables which postharvest extension and toughening usually occurred, there are limited studies on SCW deposition or the molecular biology that underpins the structure and function of the cell wall.

It has been suggested that lignification in most fruits and vegetables is also associated with lignin biosynthesis pathway, which is a complex process involving several enzymes^22,23^. Cai *et al*.^22^ observed increases in lignin and cellulose content in loquat fruit during lignification. Liu and Jiang^24^ also reported that lignin deposited continuously during lignification of green asparagus. Song *et al*.^25^ showed that the lignification of asparagus spears likely also involves the relatively high biosynthetic metabolism of heteroxylan, which is the dominant hemicellulose during this period of storage. Luo *et al*.^4^ reported that lignin biosynthesis enzymes such as phenylalanine ammonialyase (PAL), cinnamyl alcohol dehydrogenase (CAD) and peroxidase (POD) in bamboo shoot. Gao *et al*.^26^ analyzed the primary function of *PePAL* in *Phyllostachys edulis*. Peng *et al*.^27^ further characterized the genes involved in the synthesis of cellulose and lignin in bamboo shoots at the transcriptomic level. Although some lignification-associated genes, such as *PePAL, PeCAD, PeCesA* have been identified in bamboo shoots^28,29^, a comprehensive description of the MBS transcriptome related to SCW deposition remains unavailable, and the molecular mechanism underlying lignification in this context has not been fully elucidated. With the genome sequence of moso bamboo available^30^, it is feasible and reliable to identify and determine the molecular mechanisms of functional genes related to SCW deposition in the MBS.

Transcriptome sequencing is a powerful tool to rapidly obtain information on the expressed fraction of a genome, including information on gene expression, gene regulation, and the alterative splicing variance of the genes. Therefore, transcriptomic analysis is essential for interpreting the functional elements of the genome and revealing the molecular constituents of cell walls in moso bamboo^27,31^. To investigate maturation-related changes in cell wall composition and the molecular mechanism underlying maturation-related changes in the MBS cell wall, three cell wall preparations from the basal, middle and apical sections were used for comparative transcriptomic analysis to examine the molecular mechanisms underlying cell wall changes in harvested MBS. Totally, 14,902 differentially expressed genes were identified were generated from three transcriptome libraries including apical, middle, and basal segments of the bamboo shoot. Also, we have identified functional genes encoding biosynthesis of lignin, cellulose and xylan and NAC-MYB-based transcription factors. It is the first time that the molecular basis for dissecting metabolic pathways involved in the lignification have been comprehensively characterized in harvested MBS. It may provide further guide for using genetic engineering to modify lignified plant cell wall in fruits and vegetables after harvest.

## Results

### Chemical analysis of cellulose, hemicellulose and lignin in three different segments from freshly harvested MBS

Cell wall composition in the apical, middle and basal regions of MBS shoots at harvest is shown in Table 1. Lignin and cellulose contents increased from apex to base whereas hemicellulose showed a decreasing trend, and the highest level of hemicellulose was in the apical segment (Table 1). Furthermore, by comparing cell wall polysaccharides, the major component of heteroxylan was found to be 1,3,4-xylan. The total heteroxylan content decreased from apex to base, whereas 1,4-xylan increased significantly in mature sections. The highest content was observed in the basal section (Table 2). Strikingly, type I AG and type II AG arabinan levels, the main pectin polysaccharides, were higher than homogalacturonan, and showed a decreasing trend from apex to base (Table 2). Another major component of cell wall polysaccharides was found to be β-(1,3;1,4) mixed-linkage glucan (MLG), which exhibited an increasing trend from apex to base and the highest content was observed in the basal section (Table 2).

**Table 1:** Chemical composition in apical, middle, and basal segments of MBS at harvest. Data are presented as the mean ± standard error (SE) (n = 3). The error bar is 95% confidence interval and different letters indicate significant differences between apical, middle and basal segments of MBS (*P* ≦ 0.05).

Sample	Cellulose (%)	Hemicellulose (%)	Lignin (%)
Apical	19.71 ± 2.08^a^	42.43 ± 0.27^a^	10.28 ± 0.77^a^
Middle	24.10 ± 1.12^a^	35.57 ± 0.90^b^	11.85 ± 0.42^b^
Basal	29.50 ± 1.13^b^	33.71 ± 1.28^c^	13.86 ± 0.52^c^

Note: lignin as acid soluble lignin.

**Table 2:** Glycosyl linkage composition in apical, middle, and basal segments of moso bamboo shoot. Each data represents the mean of double measurements.

	Monosaccharide linkage	Apical (%)	Middle (%)	Basal (%)
Xyloglucan	1,4,6-Glc (p)	3.9	3.3	2.1
	1,4-Glc (p)	3.9	3.3	2.1
	1,2-Xyl (p)	0.3	0.4	0
	t-Xyl	0.8	0.7	0.7
	**Total Xyloglucan**	**8.9**	**7.7**	**4.8**
Heteroxylan	1,4-Xyl (p)	3.1	3.5	11.8
	1,2,4-Xyl (p)	1.4	1.4	1.3
	1,3,4-Xyl (p)	12.6	11	8.2
	1,2,3,4-Xyl (p)	2.7	2.7	2
	1,3-Ara (f)	0.9	1	0.7
	t-Ara	18.2	16.3	11.2
	**Total Heteroxylan**	**38.9**	**35.8**	**35.3**
Pectic Polysaccharides	1,5-Ara (f)	7.3	5	3
	1,3,5-Ara (f)	0.8	1.2	0.6
	**Total Arabinan**	**8.1**	**6.2**	**3.5**
	1,4-Gal (p)	5.5	5.9	3.8
	**Total Type I AG**	**5.5**	**5.9**	**3.8**
	1,3,6-Gal (p)	6.5	4.2	1.8
	**Total Type II AG**	**6.5**	**4.2**	**1.8**
	1,4-GalA	1.4	0.7	0.7
	**Total Homogalacturonan**	**1.4**	**0.7**	**0.7**
MLG	1,3-Glc (p)	1.8	3.4	5.1
	1,4-Glc (p)	4.1	7.8	11.9
	**Total 3,4-glucan**	**5.9**	**11.2**	**17**
	**Total**	**93**	**93**	**94.6**

**Figure 1:**
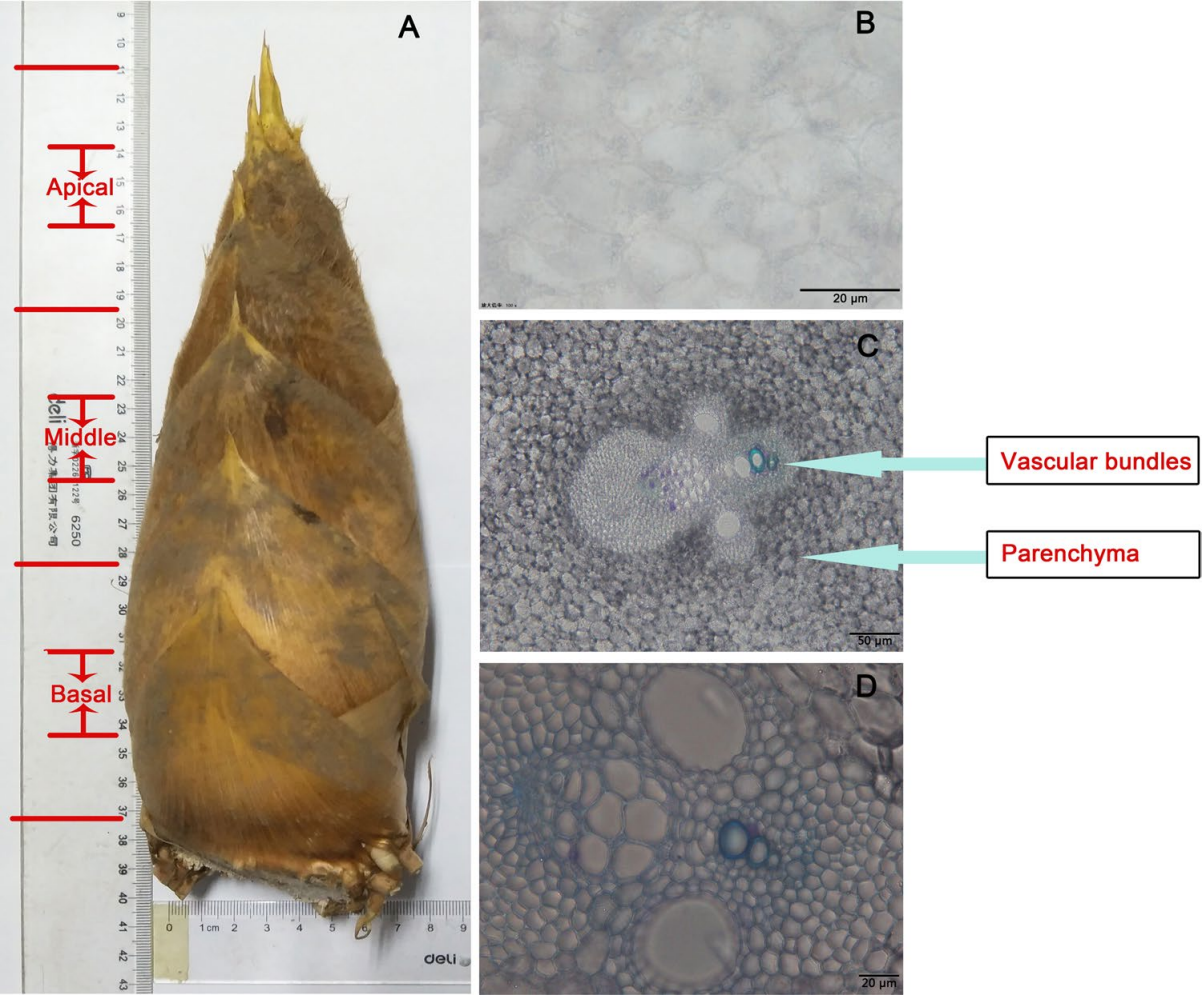
Transverse sections in the apical, middle and basal sections of MBS at harvest. (**A**) the whole shoot; (**B**) the apical; (**C**) the middle; (**D**) the basal; Scale bar: 20 μm.

Furthermore, transverse sections were taken from the three regions of MBS at harvest, where the continued development of vascular tissues involved natural wall thickening (secondary wall development) (Fig. 1). LM10 and LM11 monoclonal antibodies were used for immunolocalization of xylan in shoot sections. LM10 binds with low-substituted xylan, whereas LM11 recognizes both unsubstituted xylan as well as arabinoxylan^32^. In the present study, only LM10 labelling is shown, because of similar results obtained with the LM11 antibody (Fig. S1). The immunostaining of cross sections of shoots revealed strong signals in the walls of interfascicular fibers and xylem cells. The intensity of fluorescence signals increased drastically with increasing maturity from the apical, middle and basal regions (Fig. S1). Monoclonal antibodies JIM5, JIM7 and CCRC-M14, which bind to pectin low and high esterified homogalacturonan (Me-HG) and rhamnogalacturonan I (RG-I), respectively^33^, were used to assess changes in pectin types in the three shoot regions of MBS. As shown in Fig. S2, the strongest signals from JIM7 and CCRC-M14 were found in the apex section whereas the signal from JIM5 was weaker in the apex section than that in the middle and basal, indicating higher levels of RG-I and high-esterified homogalacturonan in the apex section, consistent with cell wall chemical analysis.

### Cell wall composition analysis of MBS by Fourier transform infrared spectroscopy

**Figure 2:**
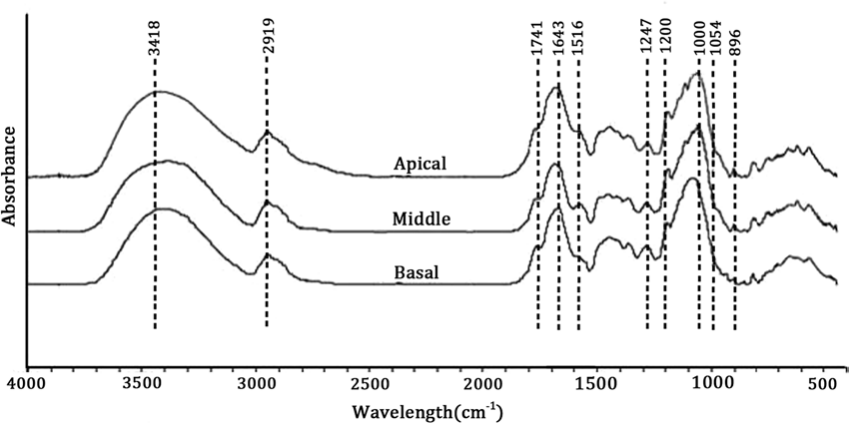
FT-IR spectra of in the apical, middle and basal sections of MBS at harvest.

In order to identify cell wall chemical changes in different sections of bamboo shoots, FT-IR was used to detect a profile for each segment based on specific spectra corresponding to specific chemical linkages^34–36^. The broad peak around 3418 cm^−1^ was assigned to the O-H hydroxyl groups (Fig. 2). The peak around 2919 cm^−1^ corresponds to hydrocarbon (C-H) stretching including the asymmetric and symmetric mode. The peaks in the fingerprint region of FT-IR spectra were ascribed as follows: 1741 cm^−1^ for unconjugated C=O in hemicellulose, 1641 cm^−1^ for absorbed water, 1546 cm^−1^ for the aromatic skeleton in lignin, and 1247 cm^−1^ for the C-O stretch in lignin. The large peak at 1020 cm^−1^ corresponded to C-O stretching vibration, whereas 898 cm^−1^ corresponded to C–H deformation in cellulose^37,38^. The 3418 cm^−1^ and 896 cm^−1^ peak represented cellulose and were the highest in basal sections, whereas the peak at 1741 cm^−1^ represented hemicellulose and was lower in the basal section than in the apical and middle segments. The peak at 1546 cm^−1^ and 1247 cm^−1^ represented lignin, and was the highest in the basal segment, which was consistent with changes in lignin content in different parts of MBS (Table 1).

### Enzymatic activities of PAL, CAD and cinnamic acid-4-hydorxylase (C4H) in the three freshly harvested segments of MBS

**Figure 3:**
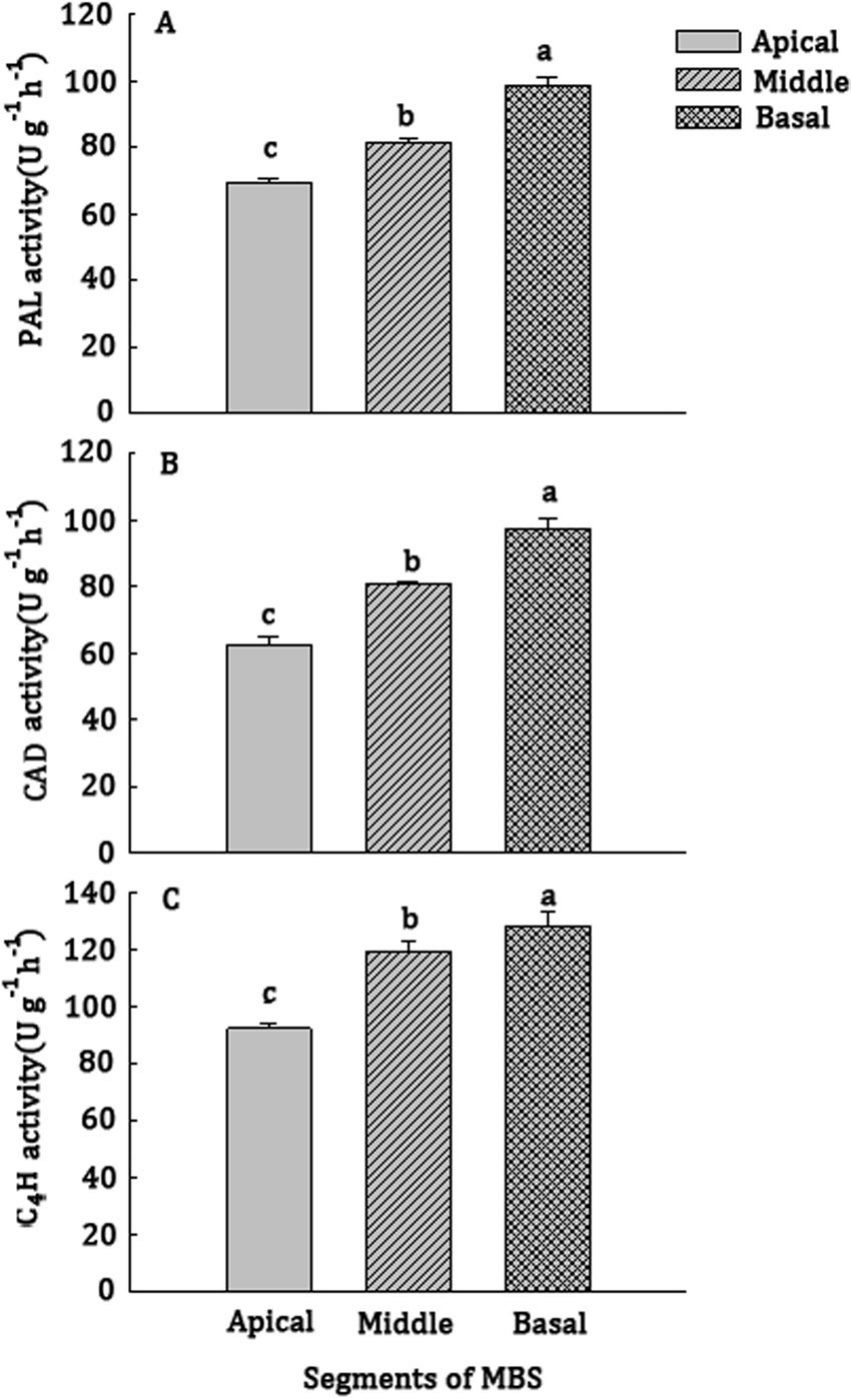
Activities of PAL (**A**) CAD (**B**) and C4H (**C**) in the apical, middle and basal segments of MBS at harvest. Data were average values ± standard error (SE) (n = 3). The error bar is 95% confidence interval and different letters indicate significant differences between apical, middle and basal segments of MBS (*P* ≦ 0.05).

Lignin is a complex polymer of phenylpropanoid mainly deposited in cell walls, which imparts rigidity to the wall, and PAL, CAD and C4H are key lignin-biosynthetic enzymes^39,40^. From the apex to base in MBS, PAL, CAD and C4H activity were shown by a very fast increase, with increased by 42.9%, 66.7% and 44.4%, respectively (Fig. 3).

### Transcriptomic analysis in the apical, middle and basal segments of MBS at harvest

To study gradient lignification of bamboo shoots, three transcriptome libraries including apical, middle, and basal segments of the bamboo shoot, were designed for high-throughput sequencing. A total of 132.9 million reads were generated, of which 48.9 million (36.79%), 41.9 million (31.52%) and 42.1 million (31.69%), respectively, were from the apical, middle and basal segments of moso bamboo shoots (Table S2). To identify the genes corresponding to these clean reads in each library, the reads were mapped to the reference genes expressed in the moso bamboo genome. Mapping results showed that 11558577 (29.42%), 9791380 (29.6%) and 9729036 (29.06%) reads from each library perfectly matched the reference genome (Table 3). Unique matched reads to the genome were 36,740,684 (93.52%), 30892394 (93.38%) and 31275082 (93.38%) in the three libraries. Only 44962902 (19.64%), 87747652 (20.96%) and 8653201 (20.54%) sequences were unmatched to the reference genome in each library, which may reflect incomplete annotation of the moso bamboo genome. The alignment statistics of these reads from apical, middle and basal segments of MBS are shown in Table 3.

**Table 3:** Alignment statistics of apical, middle and basal segments of MBS.

Tissue	Apical		Middle		Basal	
tatistical content	Number	Percentage (%)	Number	Percentage (%)	Number	Percentage (%)
Total reads	48,890,660	100	41,858,420	100	42,137,268	100
Mapped reads	39,287,568	80.36	33,083,768	79.04	33,484,067	79.46
Perfect Map	11558577	29.42	9,791,380	29.60	9,729,036	29.06
Unique mapped reads	36,740,684	93.52	30,892,394	93.38	31,275,082	93.40
Multiple mapped reads	2,546,884	6.48	2,191,374	6.62	2,208,985	6.60
Pair mapped reads	28,630,941	72.88	23,791,587	71.91	24,420,369	72.93
Single mapped reads	5,920,367	15.07	5,274,514	15.94	5,123,913	15.30
Total unmapped reads	44,962,902	19.64	87,747,652	20.96	8,653,201	20.54

### Differentially expressed genes in the apical, middle and basal segments of MBS

**Figure 4:**
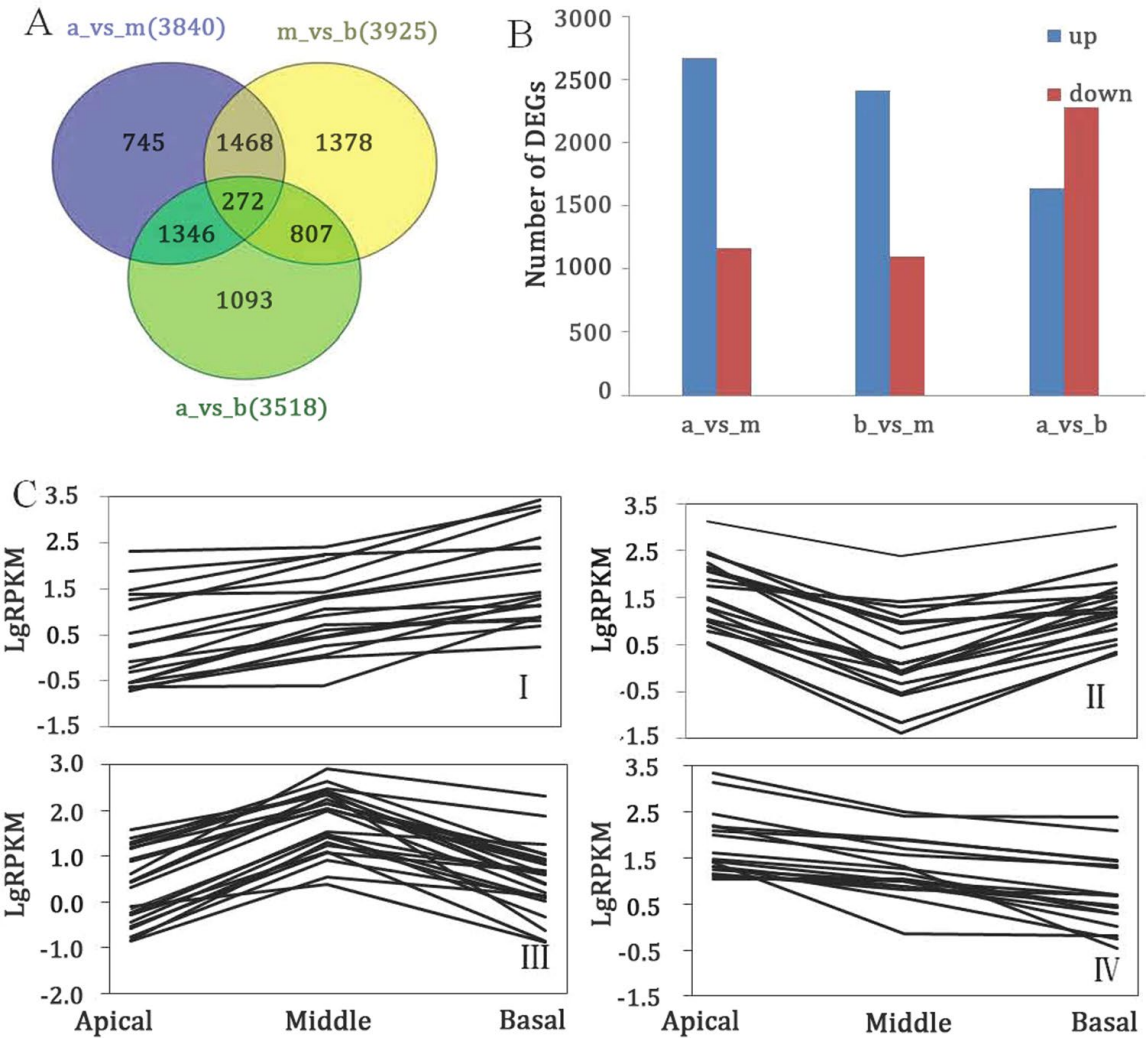
Analysis of mapped transcripts between two libraries. (**A**) Venn diagram representing the numbers of DEGs and the overlaps of sets obtained across three comparisons, where A, M and B stand for Apical, Middle and Basal, respectively. (**B**) Changes in gene expression profile among the different segments. (**C**) Patterns of gene expressions in the main clusters of three samples (Apical, Middle and Basal) by STEM analysis.

**Figure 5:**
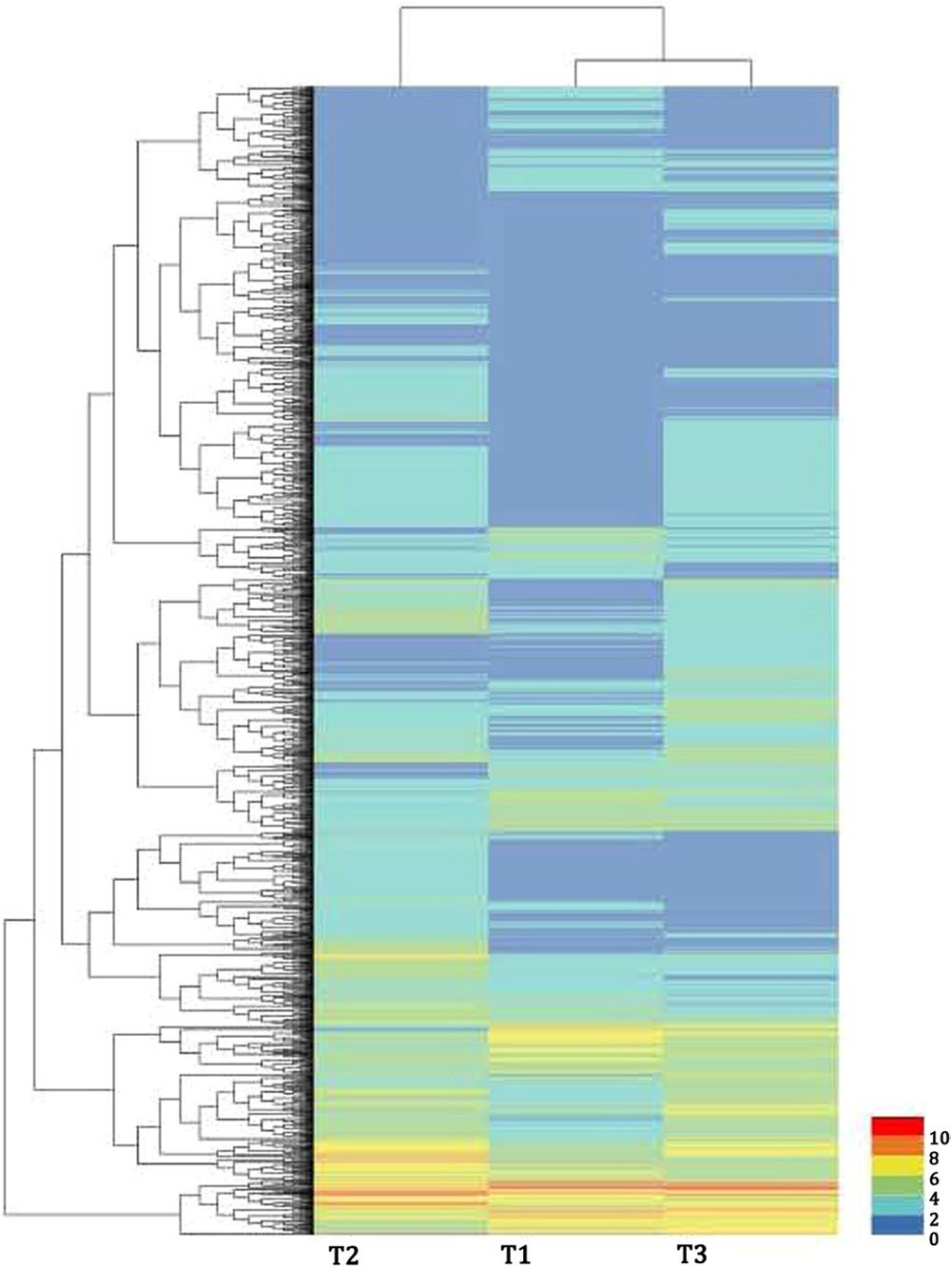
Hierarchical cluster heat map and cluster tree from DEGs between two different libraries in MBS. Heat map showed the gene expression clusters and samples clusters generated by the clustering affinity search technique (CAST) methods. Each line refers to data of one gene. The color bar represents he level of gene expression in the sample (log2FPKM + 1), ranging from blue (0) to red (10). T1: apical; T2: middle; T3: basal.

The expression abundance of each sample was measured and differentially expressed genes (DEGs) were found between each set of two libraries. First, we normalized the read density measurement and then used FDR (false discovery rates) <0.01 and the absolute value of |log2 Fold Change|≥ 1 as criteria to judge the statistical significance of gene expression. A large number of DEGs were obtained by comparing gene expression between each set of two different libraries. As shown in Fig. 4A and B, compared with each set of two sample libraries, 3840, 3518 and 3925 DEGs were predicted from “Apical vs Middle”, “Apical vs Basal”, and “Middle vs Basal”, respectively. Moreover, these DEGs were divided in to four groups according to their different expression profiles and each group contained 1303, 1756, 2992 and 1067 unigenes, respectively. Group I and group IV were defined as up-regulation, and down-regulation, respectively. Group II were shown by a decrease and followed by a fast increase, whereas group III had exhibited an inverse tendency compared to group II (Fig. 4C). These unigenes groups are shown in Table S2. The gene expression profiles are shown in a heat map (Fig. 5). Moreover, enriched genetic annotation for DEGs was analyzed, and the Cluster of Orthologous Groups (COG), Gene ontology (GO), Kyoto Encyclopedia of Genes and Genome (KEGG), Swissprot, TrEMBL, NCBI non-redundant protein (Nr), and NCBI nucleotide sequences (Nt) databases were annotated to describe the functions and metabolism of the genes as compared to the transcriptome database (*P* ≤ 0.05, hypergeometric test).

### GO annotation and KEGG pathway category

Expressed MBS genes were searched against the GO database to categorize standardized gene functions (Fig. S3). We acquired about 14902 genes that were categorized into the three main categories, which were then summarized into 44 sub-categories based on GO classification, with the categories falling under biological process (4928, 33.07%), molecular function (4608, 30.92%) and cellular component (5366, 36.01%). In the cellular component category, the most represented category was cell (GO: 0005623, 5081, 94.69%) and cell part (GO: 0044464, 5184, 96.61%), followed by organelle with 4712 genes (GO: 0043229, 88.31%). Within molecular functions, catalytic activity (GO: 0003824, 2839, 61.61%) and binding (GO: 0005488, 3229, 73.89%) were most highly represented, followed by transporter activity (GO: 0005215,421, 9.14%). For biological processes, cellular process (GO: 0051179, 4353, 88.33%) and metabolic process (GO: 0008152) with 4149 genes and 84.19% were the most prominent, indicating that these unigenes were involved in some important metabolic activities in MBS. Response to stimulus (GO: 0051234, 2863, 58.10%) was the third most highly represented category of unigenes (Fig. S3, Table S3).

To understand the biological classification and pathways that might be active in MBS, the unigenes were compared against the KEGG database. A total of 5672 genes were mapped into 107 KEGG pathways, which represent compound biosynthesis, utilization, metabolism and degradation. A summary of the genes involved in these pathways has been included in Table S4. According to our analysis, the most represented categories included ribosome (ko03010, 366 genes, 6.45%), plant hormone signal transduction (Ko04075, 273 genes, 4.81%), RNA transport (Ko03013, 271 genes, 4.78%), protein processing in endoplasmic reticulum (ko04141, 258 genes, 4.55%), starch and sucrose metabolism (ko00500, 191 genes, 3.37%), and phenylpropanoid biosynthesis (ko00940, 89 genes, 1.57%).

### Functional genes involved in lignin biosynthesis in MBS

**Figure 6:**
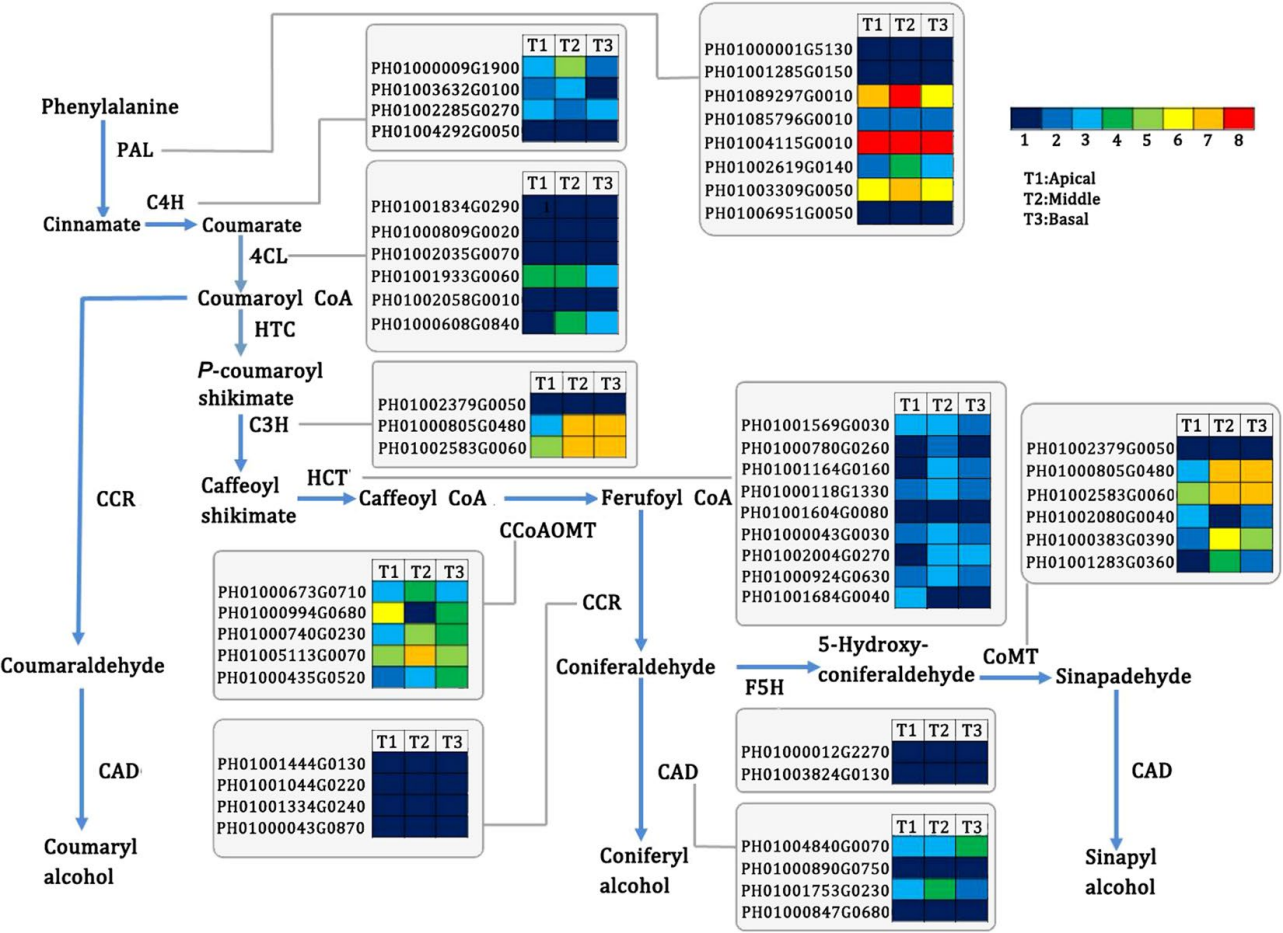
Genes involved in lignin biosynthesis in the apical, middle and basal sections of MBS at harvest.Enzyme names, unigene ids and expression patterns are indicated at each step. Grids with 8 different colors from blue to red show the RPKM values. 0–10, 10–20, 20–40, 40–80, 80–160, 160–320, 320–640 and over 640 represented by colors 1 to 8, respectively.

63 functional unigenes involved in the phenylpropanoid pathway were identified and the high expression levels of these unigenes as related to lignin biosynthesis are shown in Fig. 6 and Table S5. Genes encoding PAL, caffeic acid 3-O-methyltransferase (COMT), caffeoyl-CoA 3-O-methyltransferase (CCoAOMT) and p-coumaroylshikimate 3′-hydroxylase/coumaroyl 3-hydroxylase (C3H) showed higher expression levels than those encoding other genes. A total of 3, 2, 2, 2, 4 and 3 unigenes encoding PAL, C4H, CAD, C3H, COMT and CCoAOMT, respectively, had higher expression levels in the mature middle or basal sections than those in the immature apical section (Fig. 6 and Table S5). Also, total numbers for the 10 key genes related to lignin biosynthesis were compared in rice and *Arabidopsis*, which were identified from their respective genomes (Table 4). The number of genes encoding PAL, C4H, C3H, 4-Coumarate: CoA ligase (4CL) and Ferulate 5-hydroxylase (F5H) in our database was consistent with the moso bamboo genomic database^30^. Moreover, the number of transcripts of these 10 enzymes in MBS was more closely related to rice than to Arabidopsis (Table 4).

**Table 4:** Number of genes found in MBS transcriptome, moso bamboo genome and rice genome that encode ten key enzymes involved in the lignin biosynthesis pathway.

Enzymes	Moso bamboo	Mosobamboo^a^	Arabidopsis^a^	Rice^a^
Phenylalanine amonnialyase (PAL)	8	8	4	9
Cinnamate-4-hydroxylase (C4H)	4	4	1	4
p-Coumaroylshikimate 3′-hydroxylase/Coumaroyl 3-hydroxylase (C3H)	3	3	3	2
4-Coumarate:CoA Ligas (4CL)	6	6	4	5
Ferulate 5-hydroxylase (F5H)	2	3	2	2
Cinnamoyl-CoA reductase (CCR)	7	3	2	2
Caffeoyl caffeoyl - CoA 3-O-methyltransferase (CCoAOMT)	5	2	1	1
Cinnamyl alcohol dehydrogenase (CAD)	7	1	2	1
Hydroxycinnamoyl - CoA:shikimate/quinatehydroxycinnamoyltransferase (HCT)	14	4	1	2
Caffeic acid 3-O-methyltransferase (COMT)	7	1	1	1

^a^The results were cited from Peng *et al*. (2013).

### Functional genes involved in cellulose and non-cellulose polysaccharide biosynthesis in MBS

**Figure 7:**
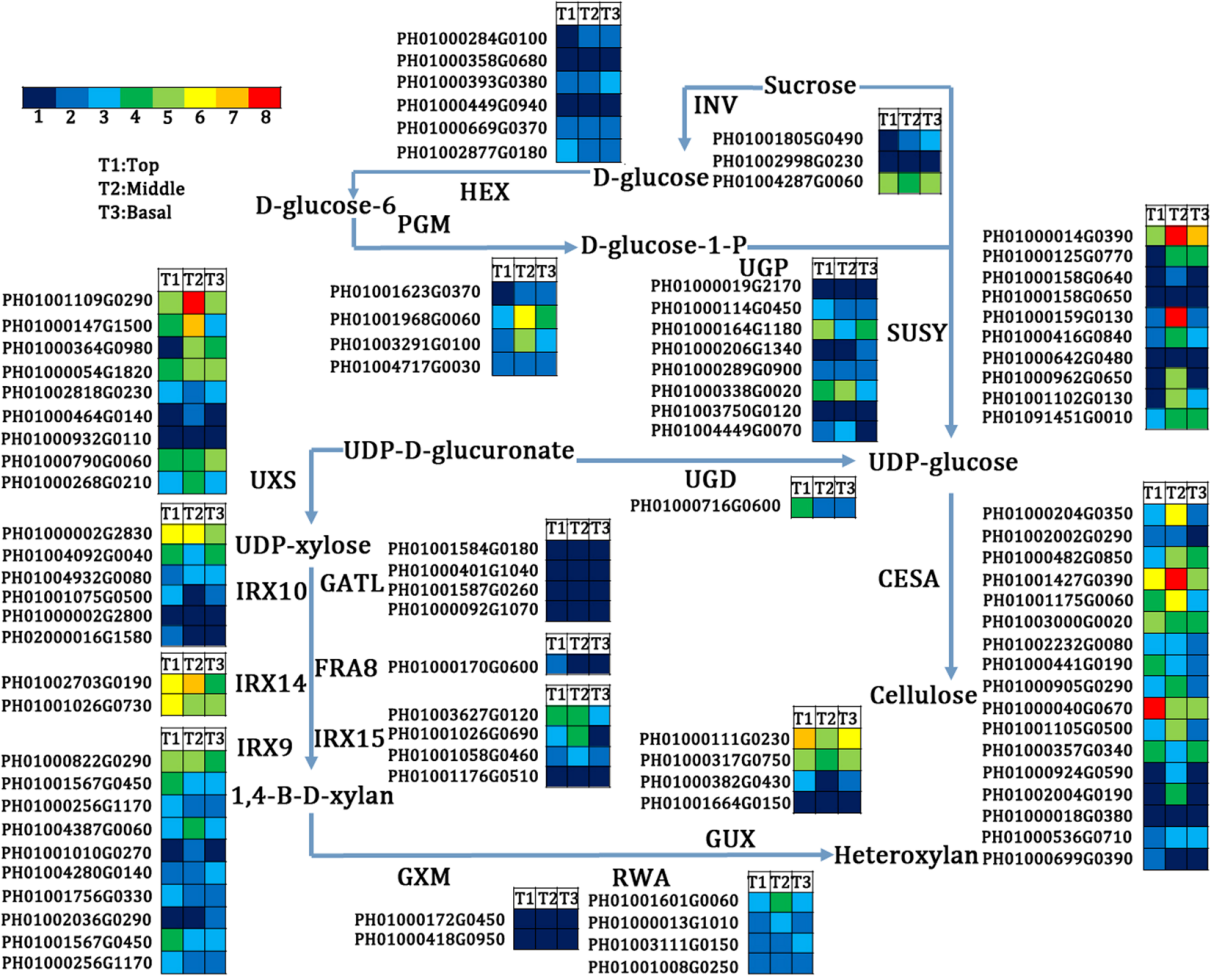
Genes involved in cellulose and xylan biosynthesis in the apical, middle and basal sections of MBS at harvest. Enzyme names, unigene ids and expression patterns are indicated at each step. Grids with 8 different colors from blue to red show the RPKM values. 0–10, 10–20, 20–30, 30–60, 60–100, 100–150, 150–250 and over 250 represented by colors 1 to 8, respectively.

The putative functional homologues of 24 genes encoding enzymes involved in cellulose and heteroxylan biosynthesis were identified (Table S5), while the expression patterns of the genes encoding the key enzymes are shown in Fig. 7. Cellulose synthase (CesA) plays a key role in regulating cellulose biosynthesis. Six unigenes corresponding to *Arabidopsis* CESA7, which was related to secondary cell wall formation, had lower expression levels, whereas CESA1 and CESA5 transcripts, related to the primary cell, were highly expressed (Fig. 7; Table S5). A total of 18 unigenes involved in the biosynthesis of xylan, which was the main hemicellulose, were identified. Among them, 2, 10 and 6 unigenes encoding *PeIRX14*, *PeIRX9* and *PeIRX10*, respectively, were identified (Fig. 7). The expression levels of IRX9 and IRX10 were observed to be higher in the middle and basal sections than in the apical section (Fig. 7).

### Functional genes involved in candidate transcription factors in MBS

**Figure 8:**
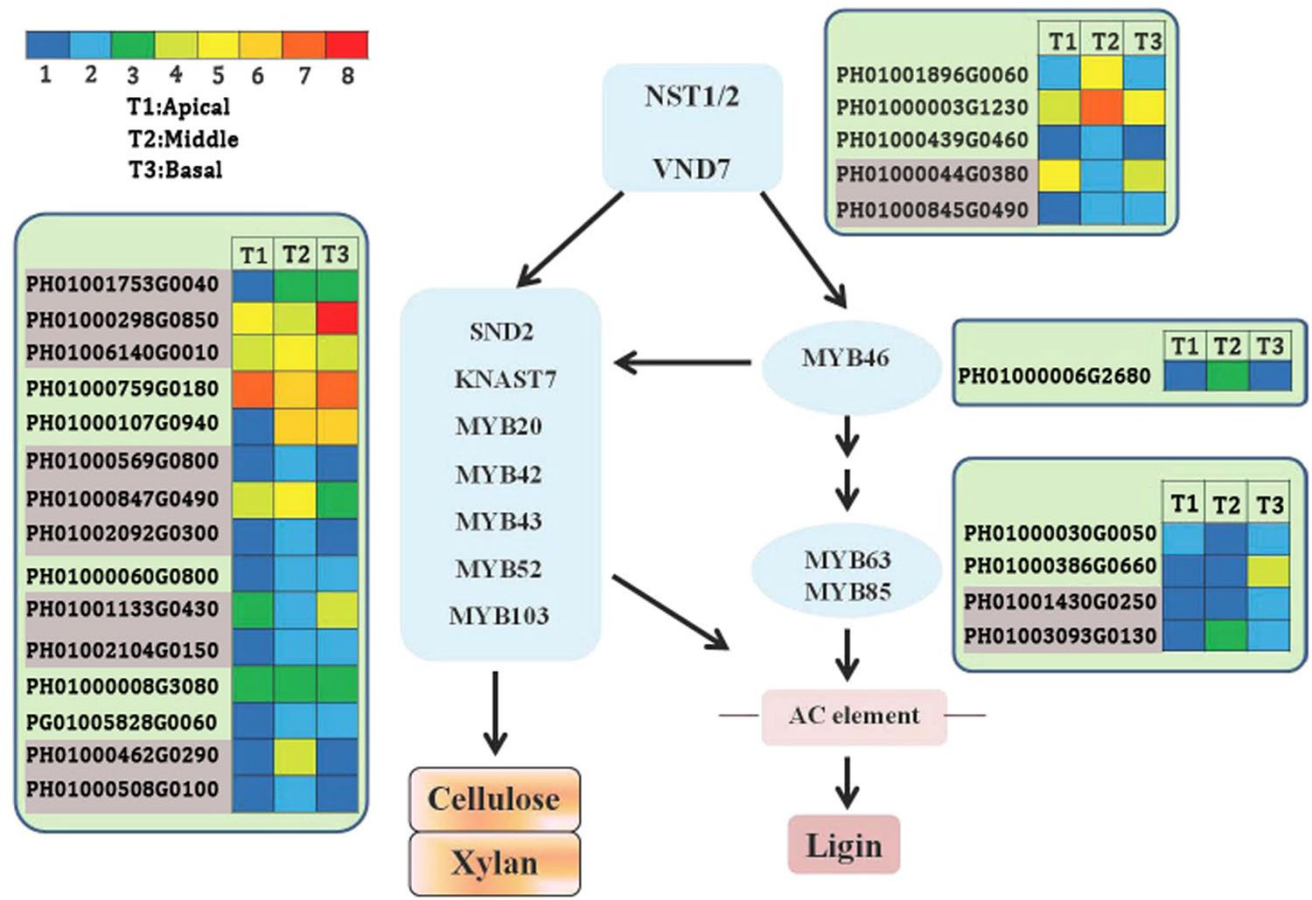
UniGenes in the transcriptional network regulating secondary cell wall biosynthesis and lignification in the apical, middle and basal sections of MBS according to *A. thaliana*. Enzyme names, unigene ids and expression patterns are indicated at each step. Grids with 8 different colors from blue to red show the RPKM values. 0–0.1, 0.1–0.5, 0.5–1, 1–2, 2–5, 5–10, 10–15 and over 15 represented by colors 1 to 8, respectively.

Transcriptional regulation is very important in regulating secondary cell wall formation in plants, including the NAC and MYB family^41,42^. In our study, 249 transcription factors of the MYB family were identified, of which 19 transcription factors mainly regulated lignin biosynthesis (Table S6). These 19 transcriptional factors were further compared with known MYBs in model plants such as Arabidopsis and rice (Table S6). One and two unigenes encoding MYB46 and MYB63, respectively, were identified and transcription analysis showed that 1 unigene encoding MYB63 had higher expression levels in the mature basal section compared to the other sections (Fig. 8). Additionally, 12 NAC transcription factors related to lignin biosynthesis were also identified from our transcriptome data, among which 1 unigene encoding NST1/2 transcription factors was observed to be higher in the mature basal and middle sections than in the apical section (Fig. 8; Table S6).

### Functional genes involved in pectin biosynthesis and metabolic regulation in MBS

The identification of genes encoding proteins that catalyze or regulate pectin synthesis and degradation is significant to understand pectin structure/function relationships. The synthesis and degradation of pectin involves a variety of enzymes that mainly includes glycosyltransferases, methyltransferases, pectin methylesterases, polygalacturonases and pectatelyases (PL). In our study, approximately 62 different unigenes encoding these key enzymes involved in pectin biosynthesis and degradation were identified in our annotated moso bamboo transcriptome database (Table S7). We also observed that expression levels of most of the gene transcripts related to pectin biosynthesis decreased, whereas those related to pectin degradation increased with shoot maturity.

### Expressional analysis of functional genes involved in cell wall biosynthesis in MBS

**Figure 9:**
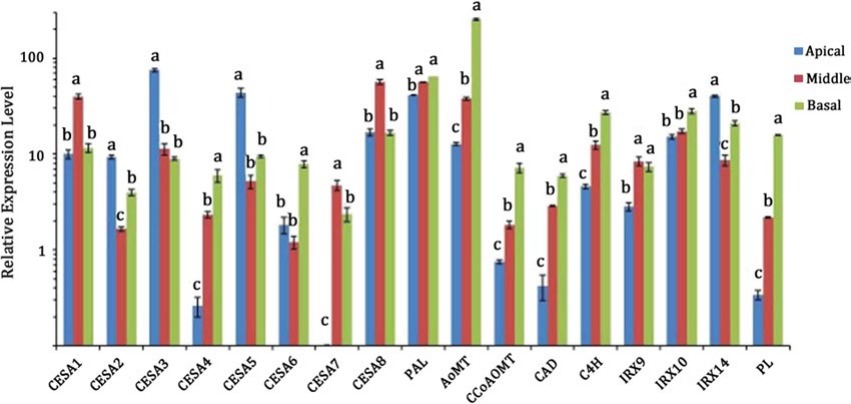
The expression profiles of the selected genes in the apical, middle and basal sections of MBS at harvest. The transcript levels of each gene were normalized to that of NTB (nucleotide tract-binding protein) at the basal section, which was set as 1.0. Each data point represents the means ± SE (n = 3). The error bar is 95% confidence interval and different letters indicate significant differences between apical, middle and basal segments of MBS (*P* ≦ 0.05).

**Figure 10:**
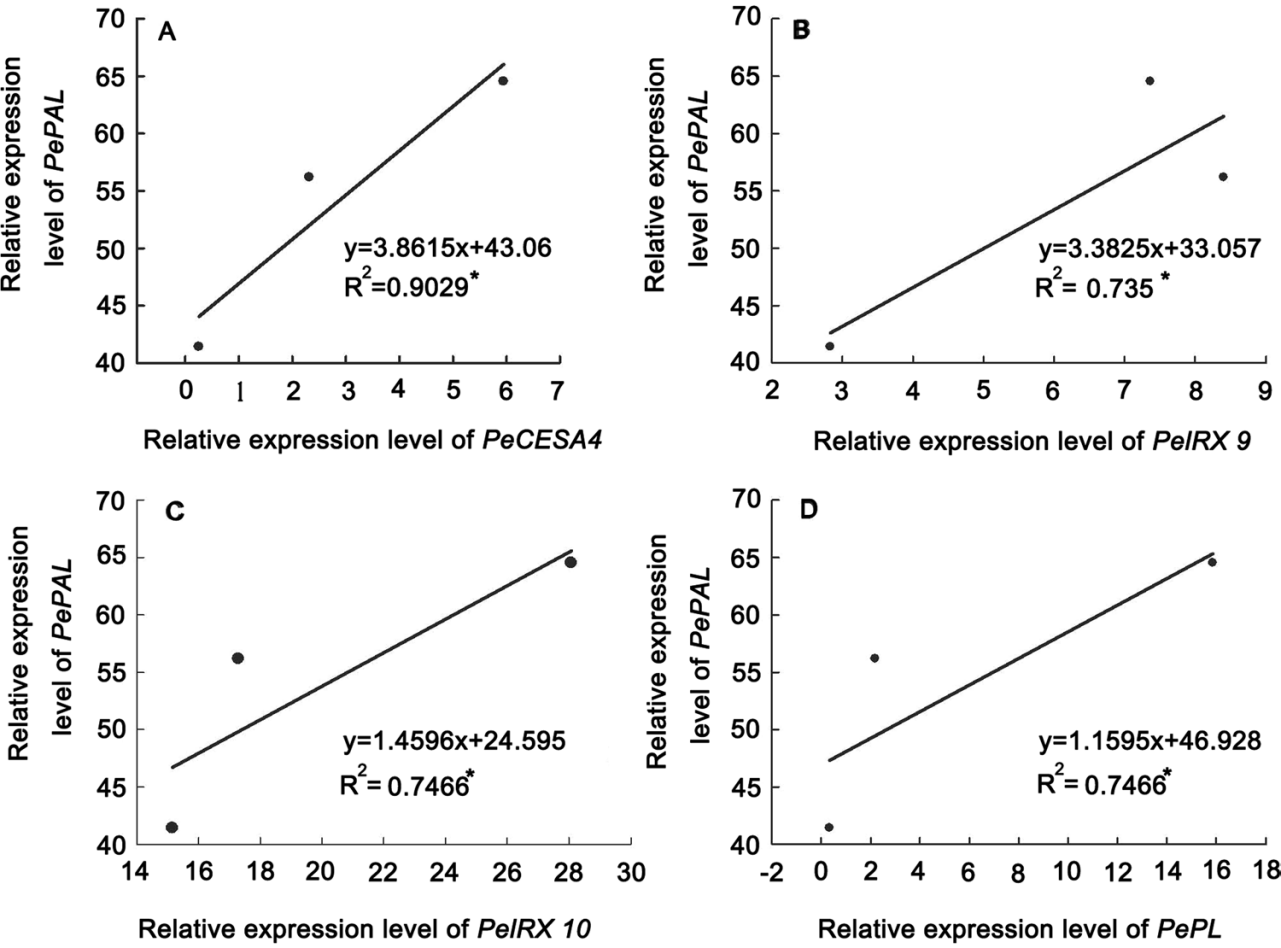
Relationship between relative expression level of *PePAL*, *PeCESA 4*, *PeIRX 9*, *PeIRX 10* and *PePL* in the apical, middle and basal sections of MBS at harvest. Each point represents the mean of 3 shoots. *means significant at *P* ≦ 0.05.

A total of 17 candidate genes were identified among those involved in cell wall biosynthesis, of which 8 genes were involved in cellulose synthesis, 5 genes in lignin biosynthesis and 3 genes in xylan synthesis (Fig. 9). Specific primers used in RT-PCR reactions were listed in Table S1 according to the open reading frames of the target genes. Expression analysis using qRT-PCR was also performed to compare the relative transcript levels of the unigenes in the apical, middle and basal segments of MBS (Fig. 9). The expression levels of the 5 genes encoding *PePAL*, *PeCoMT*, *PeCC*o*AOMT*, *PeCAD* and *PeC4H* were highest in the basal segment, and lowest in the apical segment. Interestingly, genes encoding cellulose synthase from CESA1 to CESA8 exhibited different expression patterns in the three MBS segments, among which genes encoding *PeCESA2*, *PeCESA3* and *PeCESA5* showed higher expression levels in the apical segment than those expressed in the middle and basal segments. Additionally, genes encoding *PeCESA1*, *PeCESA4*, *PeCESA6*, *PeCESA7* and *PeCESA8* exhibited higher expression levels in the middle and basal segments (Fig. 9). Furthermore, the expression of three xylan biosynthetic genes was also analyzed by RT-PCR to confirm the pattern and relative transcript abundance observed in transcriptome analysis. The expression of *PeIRX9* and *PeIRX10* in the basal and middle sections was observed to be higher than in the apical section whereas *PeIRX14* exhibited highest expression levels in the apical section among the three shoot sections (Fig. 9). Interestingly, the gene encoding PL enzyme showed the highest expression level in the most mature basal segments (Fig. 9). Furthermore, significant differences between expressions of *PePAL*, *PeCESA4*, *PeIRX9, PeIRX10* and *PePL* were analyzed, revealing positive correlation between expression levels of *PePAL*, *PeCESA4*, *PeIRX9, PeIRX10* and *PePL* with increasing maturity from the apical to basal segments in fresh MBS (Fig. 10).

## Discussion

### Maturation-related changes in the cell wall composition in MBS at harvest

In developmentally immature, rapidly growing subterraneous tissues, texture change is an important quality factor that affects post-harvest MBS ‘quality’. Xu *et al*.^3^ reported that MBS undergo a process of softening or lignifying, resulting in a loss of quality when MBS is harvested. Lignification of MBS is a significant problem that accounts for a majority of toughening-related changes and greatly influences quality after harvest^4^. This occurs even when stored at low temperature, as evidenced by increasing lignin and cellulose content. However, lignification of fibers usually does not take place homogeneously throughout the tissue^43^. In this study, the more mature basal and middle parts of the fresh MBS had higher lignin and cellulose levels than the immature apical section (Table 1), indicating that lignification increased from the apical to the base of shoots at harvest. Lignin is a complex polymer of phenylpropanoid mainly deposited in cell walls, which involves the action of several primary phenylpropanoid metabolism enzymes, as well as of lignin biosynthetic branching enzymes^44^. Increasing lignin levels in the basal and middle sections was accompanied by an increase in lignin-related enzymatic activities of PAL, CAD and C4H (Fig. 3). This further demonstrated secondary thickening and associated lignification of mature regions in fresh MBS. This result was consistent with Xu *et al*.^3^ who found that lignification took place primarily in the basal section in fresh bamboo shoots. Waldron and Selvendran^45^ also observed a progressive increase in the maturation state of tissues from the tip to the base of green asparagus spears. Conversely, the highest amounts of hemicellulose and pection polysaccharides were found in the apical segment (Tables 1 and 2). It is well known that pectin in cell walls is closely related to softening^46^. Thus, softening might occur mainly in the apical section. Similar results were reported by Femenia *et al*.^47^ in cauliflower, where the researchers found that the cell walls of the immature tissues contained more pectic polysaccharides and major hemicelluloses than mature tissues. Interestingly, although the total heteroxylan content was higher in the immature apical segment than in the mature middle and basal sections, 1,4-linked β-D-xylose (1,4-Xyl) increased from the apical region to the basal region in freshly harvested shoots (Table 2). Waldron and Selvendran^45,48^ suggested that xylan–pectic-polysaccharide complex deposition is associated with the initial stages of lignification and toughening of the stem tissues in asparagus spears. Furthermore, Awano *et al*.^49^ showed that lignin deposition occurs simultaneously with xylan penetration and microfibrils with globular xylan are masked by lignin, resulting in the homogeneous appearance of the cell wall. A previous study from our laboratory also showed that heteroxylans increased significantly in asparagus spears during cold storage^25^. Thus, high levels of lignin and cellulose in the basal section (Table 1), and heteroxylans composed of a linear backbone of 1,4-linked β-D-xylose (Xyl) residues might be related to the lignification of MBS.

### Molecular basis underlying maturation-related changes in the cell wall in MBS at harvest

Transcriptome sequencing is a relatively straight forward and precise tool for rapidly obtaining information on the expressed portion of a genome and has been increasingly used to discover genes that regulate economically siginficant traits^50,51^. By using the Illumina HiSeqTM 2500 Illumina HiSeqTM 2500 sequencing platform and selecting the moso bamboo genome database as a reference, we analyzed transcriptional changes related to secondary wall formation and lignification from the base to the top in MBS at harvest. A total of 24 functional unigenes involved in the phenylpropanoid pathway (ko00940) were ranked sixth among the top 10 pathways for DEGs between “Apical vs Basal”. Furthermore, genes encoding PAL, CoMT, CoAOMT and C3H enzyme that are involved in the phenylpropanoid and lignin biosynthetic pathways were identified^52^. The expression levels of *PePAL*, *PeCoMT*, *PeCCoAOMT*, *PeCAD* and *PeC4H*, were highest in the mature basal segment (Fig. 9), indicating key roles in lignin biosynthesis after harvest, as demonstrated by high lignin content and PAL, CAD and C4H activity in basal sections (Table 1 and Fig. 3). Luo *et al*.^4^ reported that accumulation of lignin in bamboo shoot flesh tissue during storage positively correlated to PAL and CAD activities.

CesA plays a key role in regulating cellulose biosynthesis. Zhang *et al*.^29^ suggested that *PeCesA* is involved in cellulose synthesis in secondary cell wall moso bamboo shoots. In the present study, comparative transcriptomic analysis showed that 6 unigenes corresponding to *Arabidopsis* CESA7 found in our database had lower expression levels, whereas CESA1 and CESA5 transcripts were highly expressed (Fig. 7, Table S5). This was consistent with findings by Peng *et al*.^27^, who observed that *CesA* had relatively high expression levels in the shoot. Furthermore, by expression analysis, genes encoding *PeCESA1*, *PeCESA4*, *PeCESA6*, *PeCESA7* and *PeCESA8* exhibited higher expression levels in more mature middle and basal segments (Fig. 9), which indicated that *PeCESA1*, *PeCESA4*, *PeCESA6*, *PeCESA7* and *PeCESA8* were associated with secondary cell wall deposition during lignification of MBS.

Xylan is the major hemicellulose in the secondary cell walls of eudicots and in the primary and secondary cell walls of grasses and cereals. The biosynthesis of xylan requires multiple type II glycosyltransferases (GTs) including xylosyltransferases (XylTs) responsible for generating the β-(1,4)-Xyl backbone and glucuronosyltransferases (GlcATs) and arabinosyltransferases (ArATs) responsible for incorporation of the major side chain residues^53^. In Arabidopsis, several GTs involved in heteroxylan biosynthesis have been identified via mutant analysis, which include members of the CAZy GT family 47 (*IRX10* and *FRA8/IRX7*)^10,14,54^, GT43 (*IRX9* and *IRX14*)^12,55^ and GT8 (*IRX8*, *PARVUS* and *GUX*)^12,13^. In our previous study, RNA-seq analysis was used to identify putative asparagus orthologs of known Arabidopsis xylan synthesis genes including GTs (*IRX9*, *IRX10*, *IRX14*, *FRA8*, *F8H*, *IRX8*, *PARVUS*, *GUX1*) and non-GTs (*UXS*, *GXM*, *IRX15*, *TBL29*)^25^. Of these, IRX9, IRX10, IRX14-L, IRX7, PARVUS exhibited high expression levels in the mature basal and middle sections compared to the immature apical parts^25^, indicating their roles in the lignification of asparagus spears. In the present study, 18 unigenes involved in the biosynthesis of xylan were identified and the expression of *PeIRX9* and *PeIRX10* in the basal and middle sections was observed to be higher than in the apical section (Figs 7 and 9). Thus, accompanied by high content of 1,4-Xyl in the maturation-related basal section, *PeIRX9* and *PeIRX10*, which are essential for the elongation of the xylan backbone, might play important roles in the lignification of MBS after harvest. Similarly, Suzuki *et al*.^56^ showed dense localization of xylan substitution in lignified walls in bamboo culms and indicated a close correlation with maturation and lignification of bamboo.

Furthermore, formation of secondary wall requires coordinated transcriptional regulation of genes involved in the biosynthesis of major secondary wall components (e.g. cellulose, hemicellulose, and lignin), which includes NAC and MYB transcription factors^41^. In the present study, 19 MYB and 12 NAC transcription factors were identified (Table S6). In addition, 12 NAC transcription factors were also identified (Fig. 8, Table S6), among which 1 unigene encoding NAC SECONDARY WALL THICKENING PRO-MOTING FACTOR1 (NST1)/2 transcription factors was observed to be higher in the mature basal and middle sections than in the apical section (Fig. 8; Table S6). Mitsuda *et al*.^57^ reported that NST1and NST2 regulated secondary wall thickening in Arabidopsis. Subsequent work showed that NST1 functions as a master switch of fiber cell differentiation in Arabidopsis^18,19^. Interestingly, one unigene encoding MYB63 was identified and had high expression levels in the mature basal section in freshly harvested MBS (Fig. 8). In Arabidopsis, the overexpression of MYB63 was found to induce ectopic deposition of lignin but not cellulose and xylan, whereas the dominant repression resulted in a reduction in secondary wall thickening and lignin deposition^58^. Thus, our results indicate that the NAC-MYB-based transcriptional regulatory system might modify secondary cell wall biosynthesis and is involved in the regulation of lignin biosynthesis of MBS.

Pectic polysaccharides include HG, xylogalacturonan (XGA), apiogalacturonan, rhamnogalacturonan I (RGI), and rhamnogalacturonan II (RGII)^59^. In addition to lignifying, MBS undergoes softening after harvest as demonstrated by decreasing pectin polysaccharides in the maturation-related basal section, while the ratio of HG, RGI, Arabinan and RGII showed a decreasing trend from the apical to basal section of the shoots (Table 2). It is well known that pectin metabolism includes synthesis and degradation, involving several enzymes such as glycosyltransferases, methyltransferases, pectin methylesterases, polygalacturonases and pectatelyases (PL). The identification of genes encoding proteins that catalyze or regulate pectin synthesis and degradation is important to better understand pectin structure/function relationships. In our study, 62 unigenes encoding these key enzymes involved in pectin biosynthesis and degradation were identified in the annotated MBS transcriptome database (Table S7). We also observed that expression levels of most of the gene transcripts related to pectin biosynthesis decreased, while those related to pectin degradation increased with increasing maturity in tissues from the top to the base. Interestingly, *PePL* exhibited higher expression levels in the mature basal and middle segments than in the immature apical segment (Fig. 9). Plant PLs (EC 4.2.2.2), which belong to polysaccharide lyase family 1 (PL1) (www.cazy.org), cleave the α-1,4glycosidic bond between the galacturonic acid units of HG by β-elimination and release unsaturated oligogalacturonides. Biswal *et al*.^60^ showed that the highest expression of *PtxtPL1-27* in developing secondary xylem by real time RT-PCR analysis. Thus, it is possible that *PePL* plays an important role in the secondary wall deposition and lignification of MBS after harvest. However, this observation needs to be further elucidated in the future. Furthermore, *PePAL* gene expression had significant relationships with *PeCESA4*, *PeIRX9*, *PeIRX10* and *PePL* and positive correlations between *PePAL* and *PeCESA4*, *PeIRX9, PeIRX10* and *PePL* (Fig. 10). Thus, with increasing maturity from the apical to the basal segments, secondary wall deposition and lignification of MBS closely correlated with higher expression levels of *PeCESA4*, *PeIRX9, PeIRX10* and *PePL* genes.

## Conclusion

We have analyzed maturation-related changes in the cell wall in MBS at harvest. Lignin and cellulose content increased, whereas total heteroxylan and pectin polysaccharides levels decreased with increasing maturity from the apical to basal segments. By comparing transcriptomic analysis, we have identified functional genes encoding biosynthesis of lignin, cellulose and xylan and NAC-MYB-based transcription factors, suggesting these genes closely correlated with secondary cell wall formation and lignification in MBS. Also, unigenes encoding key enzymes involved in pectin biosynthesis and degradation were identified in our annotated MBS transcriptome database. Additionally, expression analysis of candidate genes yielded further insight into the understanding of molecular mechanisms underlying maturation-related changes in the cell wall in harvested MBS.

## Materials and Methods

### Plant material

Moso bamboo (*Phyllostachysedulis*) shoots (freshly unearthed culms) with an average length of about 22 cm and an average maximum basal section diameter of 4 cm were harvested from Lin’an in Zhejiang province and transported on ice to the laboratory within 6 h post-harvest. Shoots were selected on the basis of uniformity of shape, colour, size and absence of any blemishes or disease. The outer leaf sheaths were carefully peeled off manually prior to treatment. About 3 cm was removed from the cut end of each shoot with a sharp kitchen knife.

### Treatment of apical, middle and basal segments in MBS at harvest

Processed stems were marked at 6 cm intervals and labeled basal, middle or apical according to the method described by Xu *et al*.^3^ with some modification. Samples were collected from the middle of the three sections and measured to obtain cell wall composition and lignin biosynthesis-related enzyme activity. For isolation of RNA, six MBS samples exhibiting a similar appearance were cut into three parts as described above and each section was used to extract RNA. Three biological replicate and 6 shoots per replicate were prepared. After extraction, RNA from each section of the basal, middle and apical stem segments was mixed and stored at −80 °C until they were used for transcriptome analysis.

### Immunolocalization

Sections (40 μm) from the three stem segments were cut using a Leica VT1000S vibratome supported by 3% (w/v) agarose. These sections were incubated with monoclonal antibodies of JIM5, JIM7, CCRC-M14, LM10 and LM11 for the immunostaining of pectin and xylan, respectively (1/20 dilution; Plant probes 2) for 1 h, and then washed five times with 0.1 M PBS buffer (0.1 M PBS and 0.5 M NaCl, pH 7.2). This was followed by incubation for 1 h with fluorescence isothiocyanate-conjugated antibodies (1/50 dilution; Jackson ImmunoResearch Laboratories, Inc.3). After washing five times, immunofluorescence was observed using a Zeiss LSM710 confocal microscope 4.

### Measurement of cellulose, hemicellulose and lignin contents

The method used for the analysis of cell wall components was adopted from a method described by Zhao *et al*.^61^ with some modifications. The three segments of bamboo shoot were dried and finely ground. De-waxing powders used for the following measurement were extracted with toluene-ethanol (2:1, v/v) in Soxhlet for 6 h at reflux (92 °C). De-waxing powders were subjected to sulfuric acid hydrolysis as specified in standard TappiT222 om-02 for acid-insoluble lignin. The acid soluble lignin can be measured by absorption of ultraviolet radiation [ε205 = 110 L (gcm)^−1^]. The de-waxing powders were delignified in sodium chlorite (pH 4.5, adjusted using acetic acid, 76 °C) for 4 h, leaving behind holocelluloses (hemicellulose and cellulose). The cellulose content was determined using the Kurschne–Hoffner method. De-waxing powders (1 g, dry weight) were hydrolyzed with nitric acid-ethanol multiple times in boiling water until the fiber whitened. The alcoholic nitric acid solution was discarded and fresh solution was added after each cycle. The nitric acid-ethanol solution was obtained by mixing one volume of 60% (w/w) nitric acid solution with four volumes of 95% absolute ethyl alcohol. After four cycles, the cellulose was washed, dried and weighed. The difference between holocellulose and cellulose content was defined as the hemicellulose content of the wood powder sample.

### Polysaccharide composition analysis of whole cell walls

Alcohol-insoluble residue (AIR) was prepared and subjected to methylation analysis for both neutral and acidic monosaccharide linkage composition using a previously described procedure^62^. Monosaccharide linkage analysis was performed on a Hewlett-Packard 6890 Gas Chromatograph with a Hewlett-Packard 5973 Mass Spectrometer (Agilent) equipped with a BPX70 column (25 m × 0.22 m minner diameter, film = 0.25 μm, SGE).

### Fourier transform infrared analysis

The KBr disk standard technique was used to prepare the three samples for infra-red measurements. Briefly, KBr pellets of the three samples were prepared by mixing 1 mg of powder with 100 mg KBr. Next, the mixture was squeezed using the “transmittance mode” on a BRUKER VERTEX 70 spectrometer 5. Mid-IR spectra were recorded from 4000 to 400 cm^−1^ and collected with a total of 32 scans for each sample.

### PAL, CAD and cinnamic acid-4-hydorxylase (C4H) activity assay

PAL activity was measured using the procedure described by Koukol and Conn^63^. Briefly, crude enzyme extract was obtained from 5 g frozen tissue powder with 15 mL of 0.1 M borate buffer (pH 8.8) containing 0.5 g PVPP. The homogenized mixture was centrifuged for 15 min at 14,000 × *g* and the supernatant was used to assay enzyme activity. The reaction mixture contained 0.8 ml supernatant, 2 mL 0.2 M borate buffer (pH 8.8), and 1 mL 0.02 M L-phenylalanine, which was incubated for 1 h at 30 °C. Finally, 0.5 mL of 6 M HCl was added to terminate the reaction. One unit was defined as the amount of enzyme that caused a change of 0.1 in OD_290_ per hour per gram at room temperature.

To assay CAD activity, about 5 g frozen tissue powder was extracted with 10 mL PBS (0.1 M, pH 6.25) containing 15 M β-mercaptoethanol, 2% PEG and 0.1 g PVP as reported by Luo *et al*.^4^. The homogenized mixture was centrifuged at 18,000 × *g* for 20 min and the supernatant liquid was collected to assay the enzyme activity. The assay mixture contained 0.2 mL of extract and 800 μL reaction mixture (10 mM NADP and 5 M trans-Cinnamic acid). One unit of CAD activity was defined as a change inOD_340_ per hour per gram at room temperature.

C_4_H activity was determined spectrophotometrically according to a modified version of the method described by Lamb and Rubery^64^. About 5 g of homogenized tissue was mixed with 15 mL extract (50 mM Tris-HCl, pH 8~9, 15 mM β-mercaptoethanol, 4 mM MgCl_2_, 2.5 mM ascorbic acid, 10 μM leupeptin hemisulfate salt, 1 mMphenylmethanesulfonyl fluoride, 0.15% PVP, 10% glycerine). Then, the homogenized mixture was centrifuged at 12,000 × *g* for 20 min at 4 °C and the supernatant liquid was used as the enzyme extract. C4H activity was assayed by incubating 0.8 mL supernatant liquid with 2.2 mL 50 mM Tris-HCl (pH 8.9) containing 2 μM trans-Cinnamic acid, 2 mΜ NADP and 5 μM G-6-P-Na_2_ for 30 min at 30 °C. Finally, 0.1 mL of 6 M HCl was added to terminate the reaction. One unit was defined as the amount of enzyme that caused a change of 0.1 in OD_340_ per hour per gram at room temperature.

### RNA extraction, library construction and RNA-seq

Total RNA of apical, middle and basal parts of the bamboo shoot were extracted with the Plant Total RNA Kit (OMEGA8). Three biological replicates were used for each part of the MBS. RNA quality was determined using a NanoDrop ND1000 (Thermo Scientific). RNA integrity was confirmed by electrophoresison formaldehyde-containing 1% agarose gels. Approximately 30 μg of total RNA from each sample (apical, middle and basal) was used for Illumina sequencing at Biomarker Technologies (Beijing, China). Total mRNA was extracted using NEB Next Poly(A) mRNA Magnetic Isolation Module (NEB, E7490) or using MICROBExpres Bacterial mRNA Enrichment Kit (Invitrogen, AM1905) (for prokaryotes). All procedures for cDNA libraryconstruction were performed using the NEB Next mRNA Library Prep Master Mix Set for Illumina (NEB, E6110) and NEB Next Multiplex Oligos for Illumina (NEB, E7500). Sequencing of the purified librarieswas carried out on an Illumina GA-II (Illumina Inc.USA).

### Data analysis

After RNA sequencing, high-quality clean reads were obtained bytrimming adapters and removing low-quality sequencing data defined as having more than 10% bases with a Q-value <20 and reads with unknown bases. All high-quality reads were aligned to the moso bamboo reference genome by a Basic Local Alignment Search Tool (BLAST)-like alignment tool (BLAT) (Kent, 2002). For functional annotation, unigenes were aligned using four public protein databases Nr, Nt, Swiss-Prot and KEGG (E-value ≤ 1.0 × 10^−5^). Moreover, Based on the NR annotation, GO classification was analyzed with Blast2GO software (version 2.3.5, http://www.blast2go.de/) with an E-value ≤ 1.0 × 10^−5^. For differentially expressed genes, RPKM (reads per kilobaseper million reads) values were used to calculate gene expression levels^65^. Consequently, statistical comparisons of RPKM values between different samples were conducted using the method described by Audic and Claverie^66^. The DESeq package was used to obtain the “base mean” value for identifying DEGs. FDR (False Discovery Rate) ≤0.01 and the absolute value of log2 ratio ≥1 were set as thresholds for determining significant gene expression differences between sets of two samples^67^. For the analysis of unigenes from moso bamboo that are related to metabolic pathway genes, unigenes were analyzed according to a search for standard gene names and synonyms in the functional annotations of the unigenes. Each search result was verified using BLAST. To facilitate access and analysis of the MBS transcriptome sequencing data, the Transcriptome Assembly Sequence Database were uploaded to NCBI with accession numbers PRJNA392761.

### Validation of RNA-seq data by quantitative real-time RT-PCR (qRT-PCR)

Unigenes related to metabolic pathway gene synthesis were selected for validation by qRT-PCR. Total RNA used for qRT-PCR analysis was extracted from the three parts of the shoot (apical, middle and basal) according to the procedures described above. Sequences of these selected genes were obtained from the moso bamboo genome database. The primer setfor each transcript was obtained using Primer Quest (http://www.ncbi.nlm.nih.gov/tools/primer-blast/) as shown in Table S1. NTB was used as the reference gene for all target genes^68^, and qRT-PCR was subsequently performed using SYBR Premix ExT aq™II (Takara) in a 20 μL volume with the Roche Light Cycler480 system (Roche Diagnostics). Each reaction was performed as follows: 95 °C for 30 s, 40 cycles of 95 °C for 5 s, and 60 °C for 34 s. All the samples were tested in triplicate, and the experiments were performed on three biological replicates to ensure reproducibility and reliability.

### Statistical analysis

All experiments in this study were done employing completely randomized designs. The data was tested by analysis of variance (ANOVA) using SPSS Version 11.0. Least significance differences (LSDs) were calculated to compare significant effects at the 5% level.

## Electronic supplementary material

Supplementary Information

Supplementary Table S1

Supplementary Table S2

Supplementary Table S3

Supplementary Table S4

Supplementary Table S5

Supplementary Table S6

Supplementary Table S7
