# Supplementary material for: Transcriptome analysis provides insights into xylogenesis formation in Moso bamboo (Phyllostachys edulis) shoot
Source: Sci Rep. 2018 Mar 2;8:3951. doi: 10.1038/s41598-018-21766-3 (PMC5834459; doi:10.1038/s41598-018-21766-3)

**Transcriptome analysis provides insights into xylogenesis formation in Moso bamboo (*Phyllostachys edulis*) shoot**

Hui Zhang^a1^, Ye-qing Ying^a1^, Jie Wang^a^, Xian-hai Zhao^b^, Wei Zeng^c^, Cherie Beahan^c^, Jun-bo He^b^, Xiao-yang Chen^b^, Antony Bacic^c^, Li-li Song^a*^, Ai-Min Wu^b **^

^a^ The Nurturing Station for the State Key Laboratory of Subtropical Silviculture, School of Forestry and Biotechnology, Zhejiang A & F University, Lin'an, 311300, Zhejiang Province, People’s Republic of China

^b^ Guangdong Key Laboratory for Innovative Development and Utilization of Forest Plant Germplasm, Guangzhou, 510642, China; College of Forest, South China Agricultural University, Guangzhou, 510642, China

^c^ ARC Center of Excellence in Plant Cell Walls, School of BioSciences, the University of Melbourne, Parkville VIC 3010, Australia

^1^Hui Zhang and Yeqing Ying contributed equally to this study

* The first corresponding author

Tel: 0086 (0)571 63732766

Fax: 0086 (0)571 63740809

E-mail address: lilisong@zafu.edu.cn

** The second corresponding author

Tel: 0086 (0)20 63719032

Fax: 0086 (0)571 63740809

E-mail address: [wuaimin@scau.edu.cn](mailto:wuaimin@scau.edu.cn)

All authors email address:

Hui Zhang: 570309050@qq.com

Yeqing Ying: yeqing@zafu.edu.cn

Jie Wang: 1226687101@qq.com

Xianhai Zhao: zhao_xianhai@126.com

Wei Zeng: zengw@unimelb.edu.au

Cherie Beahan: cheriew@unimelb.edu.au

Junbo He: hejunbo19@163.com

Xiaoyang Chen: xychen@scau.edu.cn

Antony Bacic: abacic@unimelb.edu.au

Lili Song: [lilisong@zafu.edu.cn](mailto:lilisong@zafu.edu.cn)

Aimin Wu: wuaimin@scau.edu.cn

Fig. S1 Immunolocalization of xylan in transverse stem sections using the LM10 antibody. Transverse sections of stem material from apical (A), middle (B) and basal (C) parts. Scale bar, 50 μm.

§

A

B

C


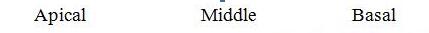

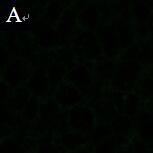

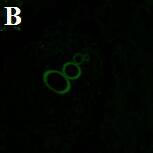

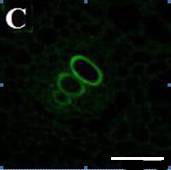


Fig. S2 Immunolocalization of pectin in transverse stem sections using JIM5, JIM7 and CCRC-M14 antibody. Transverse sections of stem material from apical (A, D, G), middle (B, E, H) and basal (C, F, I) parts. Scale bar, 50 μm.


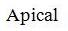

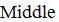

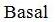


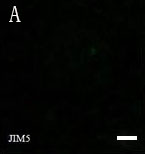

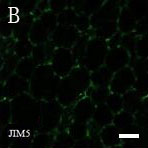

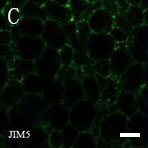


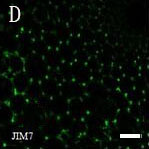

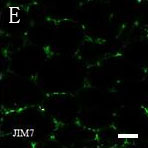

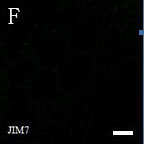


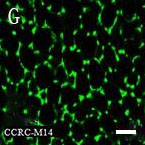

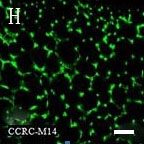

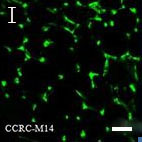


Fig. S3: GO classification of differentially expressed genes among different libraries in MBS.


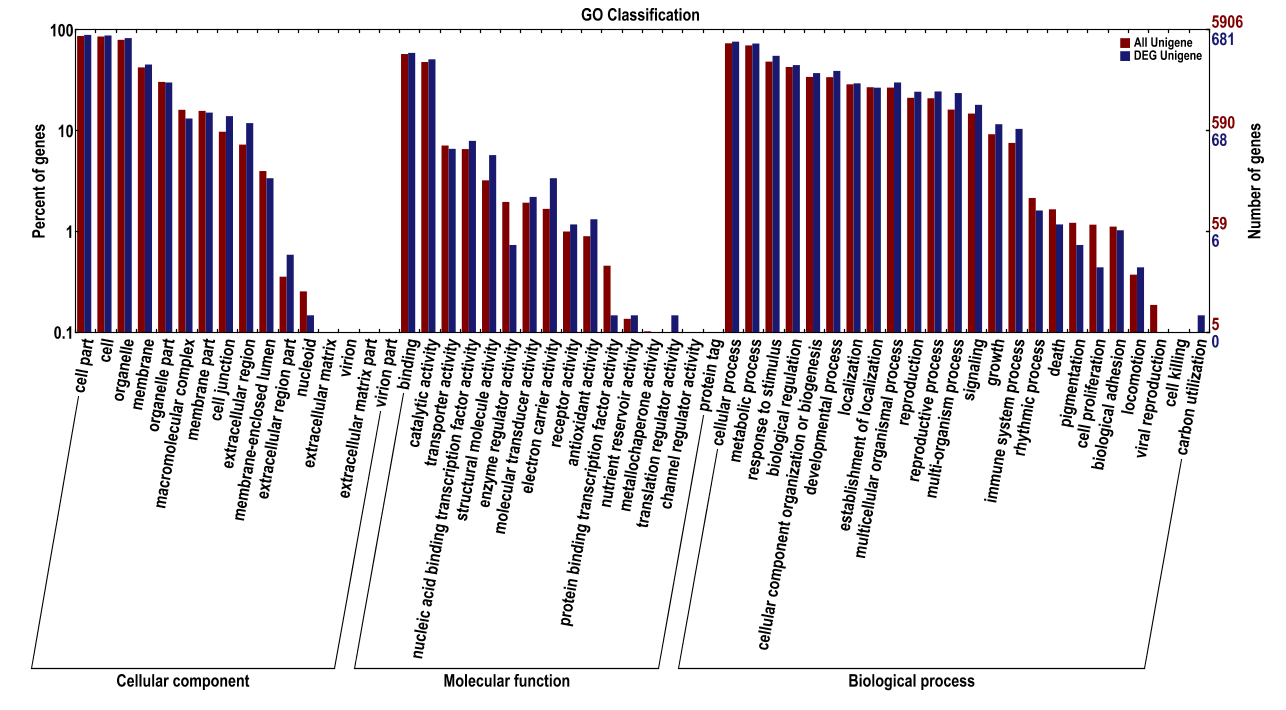


Fig. S4: Phylogenetic relationship of MYB transcription factor proteins, which might be involved in the secondary cell wall biosynthesis, from Arabidopsis, rice and moso bamboo. The whole sequences were aligned using ClustalX2.1 and a bootstrapped neighbour joining tree was constructed by MAG6.0.


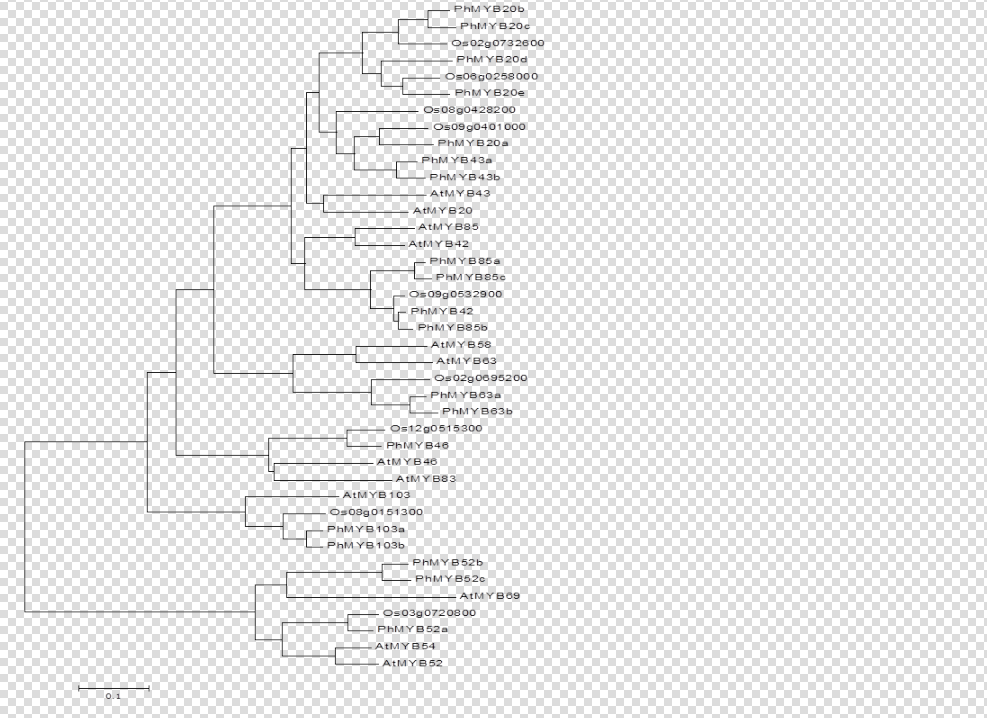

Supplement: Supplementary file 1 — Supplementary Information [file 41598_2018_21766_MOESM1_ESM.doc]
